# Supplementary material for: Resolving the full spectrum of human genome variation using Linked-Reads
Source: Genome Res. 2019 Apr;29(4):635–45. doi: 10.1101/gr.234443.118 (PMC6442396; doi:10.1101/gr.234443.118)
Supplement: Supplemental Material [file supp_gr.234443.118_Supplemental_longranger-2.2.zip › longranger-2.2.tar.bz2!/lr2.2-public/lib/stan/cmdstan/stan/doc/api/html/classstan_1_1mcmc_1_1base__adaptation.html]

Stan: stan::mcmc::base\_adaptation Class Reference


|  |  |
| --- | --- |
|  | Stan  2.5.0  probability, sampling & optimization |


- Main Page
- Related Pages
- Namespaces
- Classes
- Files

- Class List
- Class Index
- Class Hierarchy
- Class Members

All Classes Namespaces Files Functions Variables Typedefs Enumerations Enumerator Friends Macros Pages

- stan
- mcmc
- base\_adaptation

Public Member Functions |
List of all members

stan::mcmc::base\_adaptation Class Reference

`#include <base_adaptation.hpp>`

Inheritance diagram for stan::mcmc::base\_adaptation:

|  |  |
| --- | --- |
| Public Member Functions | |
| virtual void | restart () |
|  | |

## Detailed Description

Definition at line 8 of file base\_adaptation.hpp.

## Member Function Documentation

|  |  |  |  |  |
| --- | --- | --- | --- | --- |
| virtual void stan::mcmc::base\_adaptation::restart | ( |  | ) |  |

inlinevirtual

Reimplemented in stan::mcmc::stepsize\_adaptation, and stan::mcmc::windowed\_adaptation.

Definition at line 12 of file base\_adaptation.hpp.

---

The documentation for this class was generated from the following file:

- src/stan/mcmc/base\_adaptation.hpp


---

[ Stan Home Page ]

*© 2011–2014,
Stan Development Team.*
